# Gastric MALT lymphoma presented with primary perforation in an adolescent: a case report

**DOI:** 10.1186/s12887-019-1431-9

**Published:** 2019-02-19

**Authors:** Yu-Tang Chang, Ming-Yii Huang, Hsiang-Hung Shih, Chun-Chieh Wu, Tzu-Ying Lu, Pei-Chin Lin

**Affiliations:** 1 Division of Pediatric Surgery, Department of Surgery, Kaohsiung Medical University Hospital, Kaohsiung, 80756 Taiwan; 2 Department of Radiation Oncology, Kaohsiung Medical University Hospital, Kaohsiung, 80756 Taiwan; 3 Department of Pediatrics, Kaohsiung Medical University Hospital, Kaohsiung, 80756 Taiwan; 4 Department of Pathology, Kaohsiung Medical University Hospital, Kaohsiung Medical University, Kaohsiung, 80708 Taiwan; 5 Graduate Institute of Medicine, College of Medicine, Kaohsiung Medical University, Kaohsiung, 80708 Taiwan; 6 Department of Surgery, Faculty of Medical School, College of Medicine, Kaohsiung Medical University, Kaohsiung, 80708 Taiwan; 7 Department of Radiation Oncology, Faculty of Medical School, College of Medicine, Kaohsiung Medical University, Kaohsiung, 80708 Taiwan; 8 Department of Pediatrics, Faculty of Medical School, College of Medicine, Kaohsiung Medical University, Kaohsiung, 80708 Taiwan; 9 Department of Pathology, Faculty of Medical School, College of Medicine, Kaohsiung Medical University, Kaohsiung, 80708 Taiwan

**Keywords:** Gastric MALT lymphoma, Laparoscopy, Perforation, Iron deficiency, Adolescent

## Abstract

**Background:**

Primary lymphomas of the gastrointestinal tract are rare, accounting for only 1 to 4% of malignancies arising in the stomach, small intestine, or colon. The stomach is the most common extranodal site of lymphoma and gastric mucosa-associated lymphoid tissue (MALT) lymphoma accounts for 40% of primary gastric lymphoma. Gastric MALT lymphoma reaches its peak incidence between 50 to 60 years of age, therefore, it is rarely encountered in pediatric population. The presenting symptoms of gastric MALT lymphoma are usually nonspecific and primary perforation of gastric MALT lymphoma is uncommon.

**Case presentation:**

A 12 year-old female presented with iron deficient anemia developed gastric perforation. Emergency laparoscopic repair of the perforation was performed and tissue pathology showed gastric MALT lymphoma infiltration. *Helicobacter pylori* eradication and radiotherapy were sequentially performed. Complete remission was achieved at two months after radiotherapy. To our best knowledge, she is the youngest patient with gastric MALT lymphoma reported in the literature.

**Conclusion:**

Iron deficient anemia is a common presenting manifestation of malignancies in adulthood. In pediatric population, iron deficient anemia is usually caused by nutritional deficient or blood loss. In this case report, we present a teenaged female without previous gastric ulcer history who presented with a rare gastric tumor and an uncommon primary perforation. Even if there is an uncertainty about the exact diagnosis prior to the surgery, the strategy of stomach-preserving therapy by laparoscopy for primary perforation was successful and provided a good quality of life.

**Electronic supplementary material:**

The online version of this article (10.1186/s12887-019-1431-9) contains supplementary material, which is available to authorized users.

## Background

Extranodal marginal zone B cell lymphoma of mucosa-associated lymphoid tissue (MALT lymphoma), which was previously considered a low-grade lymphoma, is the predominant histological subtype of primary gastric lymphoma, representing 1–6% of primary gastric neoplasm, 5–7% of all non-Hodgkin lymphomas, 40–50% of all gastric lymphomas, and 50–60% of all extranodal lymphomas [1–4]. The common symptoms of gastric MALT lymphoma include epigastric pain, nausea, vomiting, weight loss, and gastrointestinal bleeding [2, 3]. However, the description of perforation at presentation is rare in the literature [5–7]. Herein, we present such a case with successful management in a female adolescent.

## Case presentation

A 12-year-old girl was admitted with noticeable palor and dyspnea on exertion for the past two weeks. No specific medicine or family histories were reported. She visited local clinics and her hemogram showed a low hemoglobin value. Physical examination showed a palor and mild tachycardia (110 bpm). Laboratory data taken in our hospital showed a hemoglobin level of 5.9 g/dL; mean corpuscular volume of 75.4 fl; C-reactive protein level of 1.02 mg/L; serum ferritin of 2.9 ng/mL; serum iron level of 9 μg/dL; and total iron binding capacity at 458.2 μg/dL. She denied bloody stool or abdominal discomfort history. Iron tablet (100 mg bid) was prescribed. Stool examination showed a mild hemoccult-positive (1+). ^13^C urea breath test was a positive finding. Therefore, upper GI endoscopy was arranged.

**Fig. 1:**
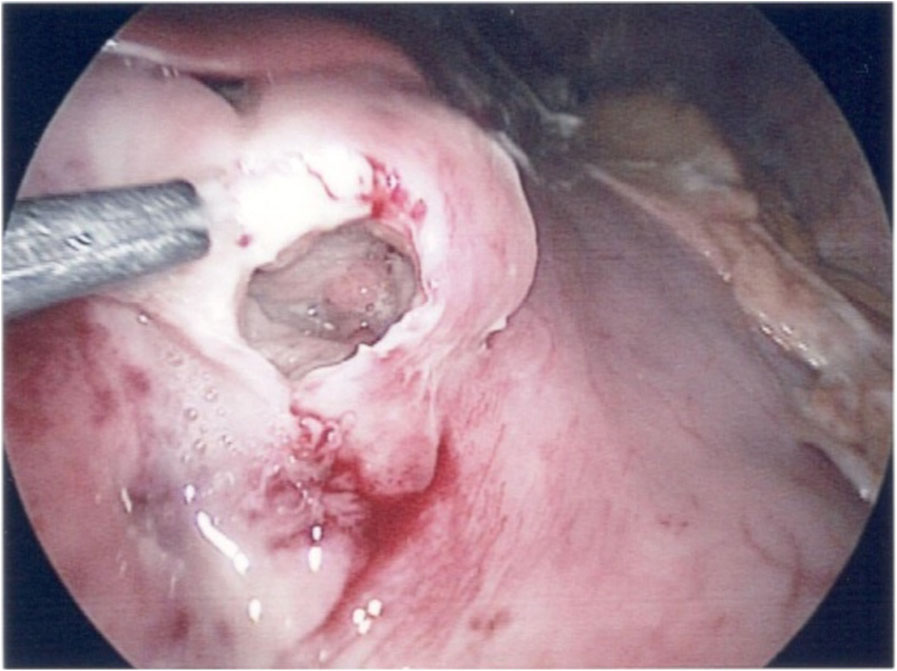
Laparoscopic finding. Note the solitary perforation over the gastric body

However, 8 h prior to scheduled exams, patient complained of sudden onset of severe tenderness with involuntary guarding and rebounding pain involving the entire abdomen. Interpretation of standing view and left lateral decubitus abdominal film detected free intraperitoneal air, and peritonitis was confirmed. Because of the abnormal image findings, surgical intervention was advised and in light of hemodynamic stability, a laparoscopic approach was performed. After initial exploration of the peritoneal cavity, a burst perforation, approximately 1 cm in diameter, was noted over lower gastric body (Fig. 1). The edge of the perforation was excised, and simple closure was performed. The resected specimen was sent for pathological examination.

**Fig. 2:**
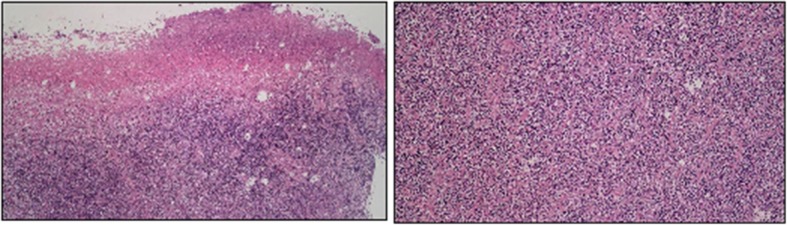
HE stain of gastric tissue. At low power field (left), the specimen shows an ulcerated surface with fibrinopruvulent and necrotic materials coated, and mixed inflammatory cells infiltrate in the deep submucosal or muscular layers. However, there are some large, atypical lymphoid cells scattered distributed in the background at high power field (right)

**Fig. 3:**
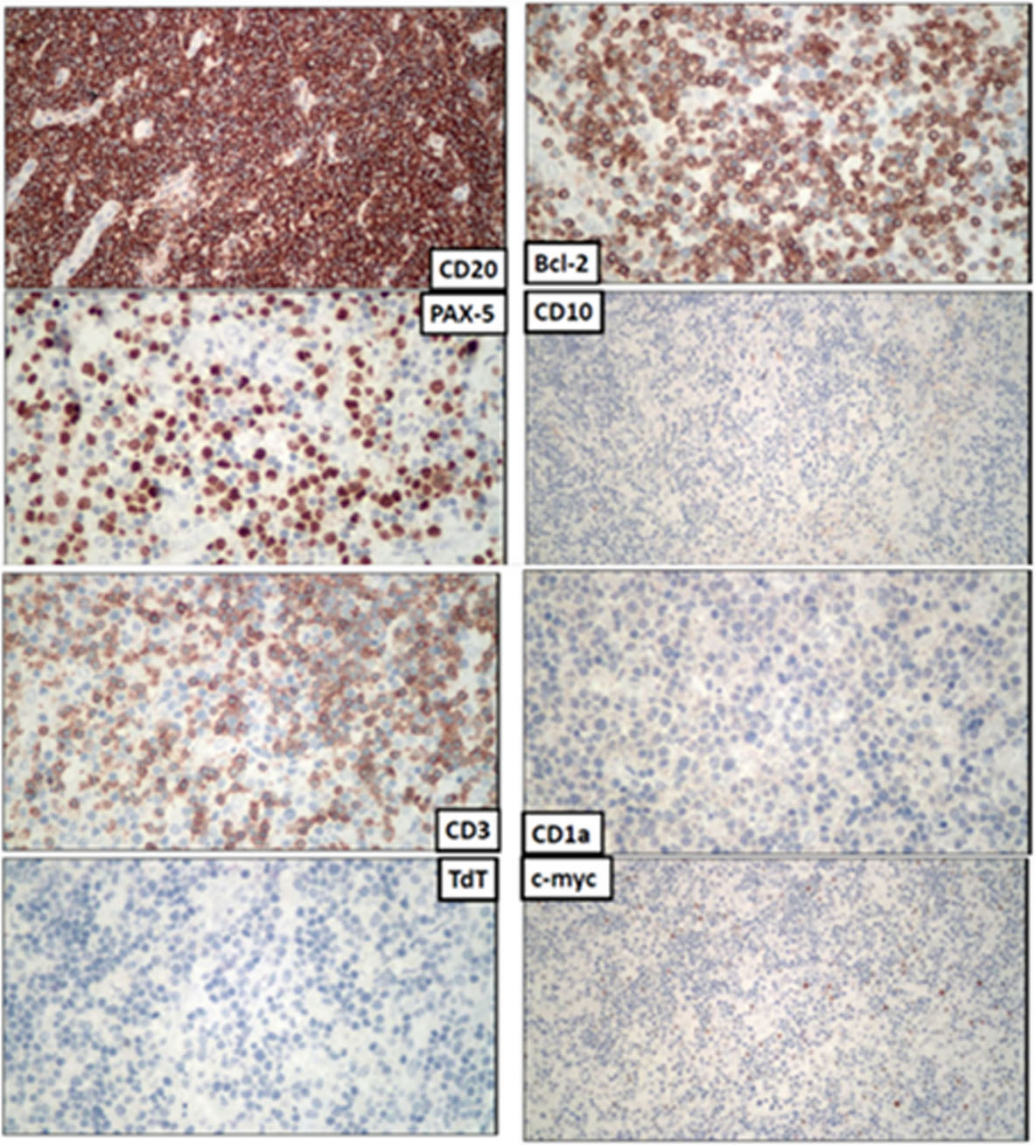
Immunohistochemistry stain of gastric tissue. These atypical lymphoid cells are immunoreactive for CD20, PAX-5, Bcl-2, and negative for CD3, CD10, CD1a, TdT and c-myc

**Fig. 4:**
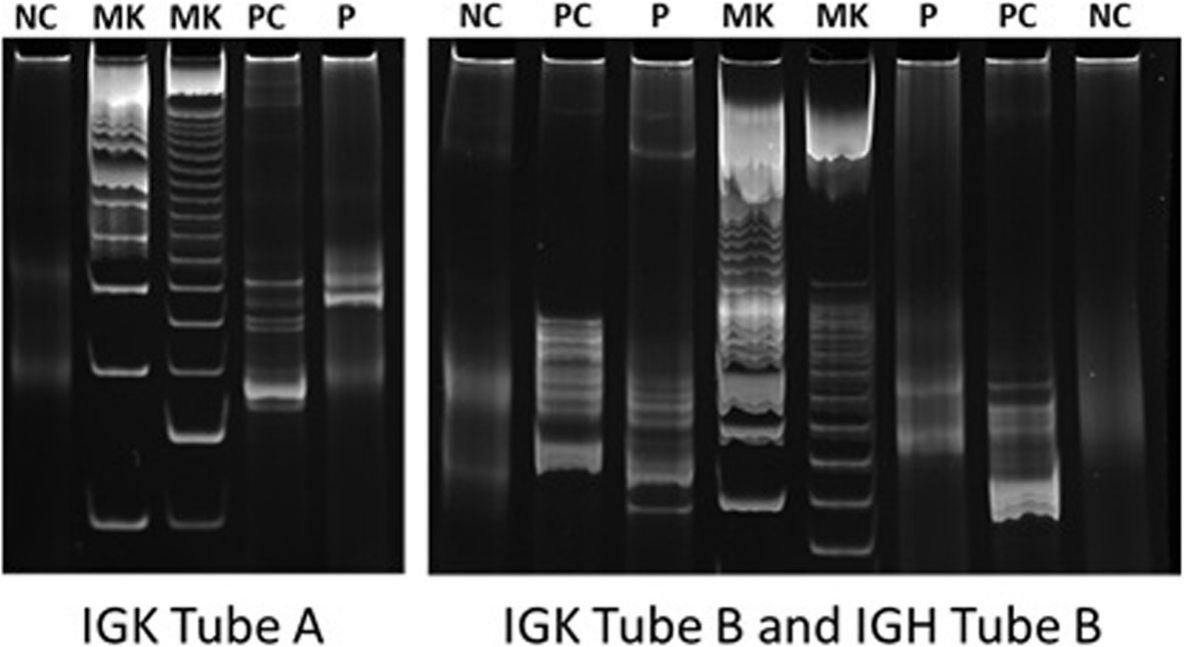
Polymerase chain reaction-based clonality study for immunoglobulin gene rearrangement. Monoclonal were detected by BIOMED2 IGK Tube A, IGK Tube B and IGH Tube B reactions. (NC: negative control, MK: marker, PC: positive control, P: patient)

Histology confirmed the diagnosis of extranodal marginal zone B-cell lymphoma of MALT type. Section showed diffuse infiltration of small lymphocytes without residual normal architecture. The aggregation of tumor cells were composed of monocytoid cells with plasmacytoid and centrocyte-like cell differentiation (Fig. 2). Immunohistochemically, these cells were positive for B-lymphocyte antigen cluster of differentiation (CD) 20, CD79a, and paired box protein Pax-5, but negative for CD3, CD5, CD10, B-cell lymphoma 2, CD30, terminal deoxynucleotidyl transferase, CD1a, c-Myc, and S100 (Fig. 3). Light-chain restriction for infiltrating plasma cells was not identified. Both Epstein-Barr encoding region in situ hybridization and cytomegalovirus were negative. The B-cell clonality exhibited monoclonality (Fig. 4).

Subsequently, a systemic workup for clinical staging, including lactate dehydrogenase (161 IU/L), β2-microglobulin (148.0 μg/dL), hepatitis B virus (nonreactive), hepatitis C virus (negative), and human immunodeficiency virus (negative), was performed. Positron emission tomography-computed tomography (PET-CT) showed accumulation of fluorodeoxyglucose in the same area. CT, bone scan, and bone marrow biopsy were also performed, and no metastatic lesion was detected. The Lugano staging system was considered to be Stage IE.

**Fig. 5:**
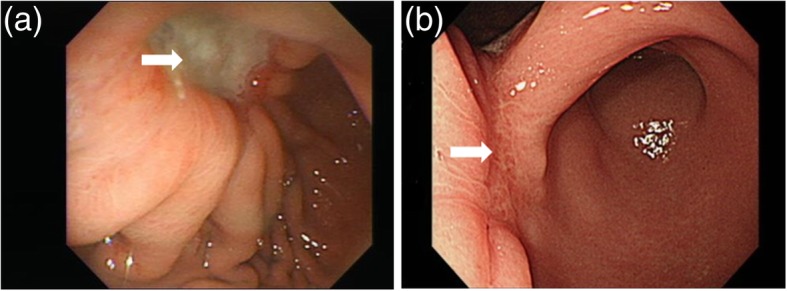
Endoscopic findings. **a** Endoscopic finding after operation. An ulcerative lesion over lower gastric body close the gastric angle can be seen. Suture line was observed at the bottom of the crater (white arrow). **b** Endoscopic finding after radiotherapy. The ulcerative lesion was healed with scar formation (white arrow)

After resuming an oral diet, a 2-weeks course of oral antibacterial treatment (clarithromycin 500 mg plus amoxicillin 500 mg twice a day for 7 days followed by metronidazole 500 mg twice a day for another 7 days) plus 4 weeks esomeprazole (40 mg daily) were prescribed for *Helicobacter pylori* infection eradication. Endoscopy was scheduled 4 weeks after operation and showed a deep and large ulcer over anterior wall of the body with convergence of thickened mucosal folds (Fig. 5a). Biopsy samples were again obtained and consistent with extranodal marginal zone lymphoma of MALT. Therefore, involved field radiation therapy was delivered to the stomach (30 Gy in 20 fractions given over 4 weeks). There were no gastrointestinal side effects noted during and after radiotherapy.

A follow-up endoscopy was performed at 4 months after operation, and showed a broad-based healed scar with rugae interruption (Fig. 5b). The histological evaluation of biopsy specimen showed absent plasma cells and small lymphoid cells and complete histological remission was achieved at 2 months after radiotherapy. During a 1-year follow-up at our outpatient clinic, she has remained free of symptoms and without relapse. The timeline was shown in Additional file 1.

## Discussion and conclusions

Since MALT lymphoma is characterized as “low-grade” (or indolent) and has a natural history of slow progression, most cases occur in individuals 50 years or older, with disease being most common in the sixth decade [2–4]. To our best knowledge, this 12-year-old girl is the youngest patient with gastric MALT lymphoma reported in the literature.

*H pylori* has been identified as the cause of chronic gastritis with consequent acquisition of lymphatic tissue, and up to 98% of gastric MALT lymphoma are second to *H pylori* infection [8]. According to the clinical practice guidelines recommended by the European Society for Medical Oncology (ESMO) Guidelines Working Group [9], *H pylori* eradication is the first-time treatment in any case irrespective of *H pylori* status and lymphoma stage [2, 8], and lead to a complete remission in 50–90% of cases [4]. Those patients revealing persistence or progression of lymphoma despite successful *H pylori* eradication should receive radiation or chemotherapy [1]. Surgery usually does not play a role in the therapy of gastric MALT lymphoma, however, complications such as perforation or bleeding that cannot be controlled endoscopically may require surgical intervention [2, 8].

The infiltration of MALT lymphoma is mostly confined to the mucosa, and only 10% of infiltration invade deeply beyond muscularis propria [4]. Therefore, primary perforation is a rare complication of gastric MALT lymphoma. On reviewing the literature starting from the first description of MALT lymphoma in 1983 [10], only 4 cases have been reported [5–7, 11]. Of these 4 patients, 3 were men, ranging in age from 24 to 84 years. Due to the rare cases reported in the literature, the management for primary perforation of gastric MALT lymphoma has been gastrectomy with lymphadenectomy [5–7]. However, in the case of low-grade lymphoma, immediate radical resection may be unnecessary and an organ-preserving therapy would provide a better quality of life [12]. Simple closure of perforated gastric MALT lymphoma (followed by clinical practice guidelines according to the ESMO Guidelines Working Group) seems to be an acceptable treatment. As experience with minimally invasive surgery has expanded in perforated peptic ulcer, laparoscopy is both feasible and safe for a gastric perforation by MALT lymphoma.

Radiotherapy usually offers a curative option to patients with *H pylori* negative or refractory to *H pylori* eradication [1]. In the present case, radiotherapy was given because of deep invasion and patient’s young age. The major concern of radiotherapy for the patient was the risk of radiotherapy-related gastric perforation and bleeding, about 4% reported in the literature [1, 13]. However, involved field radiotherapy with moderate-dose (30-Gy) may improve the target coverage and reduce radiation dose. Since gastric carcinoma is also associated with *H pylori* gastritis, 5% of metachronous gastric carcinoma occurred after remission of gastric MALT lymphoma and the risk of development of gastric carcinoma in patients with gastric MALT lymphoma were shown to be 6 times higher than in the general population [4, 14, 15]. Due to the diagnosis at the young age in the present case, long-term follow-up is mandatory for detection of metachronous gastric carcinoma at an early stage.

In pediatric population, iron deficient anemia is usually caused by nutritional deficient or blood loss. This case presents a relatively uncommon clinical problem. Even if there is an uncertainty about the exact diagnosis prior to the surgery, a stomach-preserving therapy by minimally invasive surgery is acceptable and should provide a good quality of life.

## Additional file

Additional file 1: Timeline. (PDF 650 kb)

## Abbreviation

MALT: Mucosa-associated lymphoid tissue
