# Supplementary material for: Gastric MALT lymphoma presented with primary perforation in an adolescent: a case report
Source: BMC Pediatr. 2019 Feb 19;19:63. doi: 10.1186/s12887-019-1431-9 (PMC6380025; doi:10.1186/s12887-019-1431-9)
Supplement: Supplementary file 1 — Timeline. (PDF 650 kb) [file 12887_2019_1431_MOESM1_ESM.pdf]

A 12-year-old girl was admitted to our institution in April, 2016. She was admitted with noticeably pale skin and dyspnea on exertion within the past two weeks.

6/4/2016

Laboratory data:
Hemoglobin: 5.9(11.1~15.1)g/dL; Mean corpuscular volume: 75.4(83.4~98.5) fl; RDW-CV: 17.3 % (11.7~14.7 %); Reticulocyte: 2 % (0.87~2.26 %)
Total bilirubin/direct bilirubin: 0.39/<0.1 (0.2~1/0.0~0.2) mg/dL
Serum iron: 9 ug/dL (28~170 ug/dL); UIBC: 449.2 ug/dL; TIBC: 458.2 ug/dL (254~450 ug/dL)
Ferritin: 2.9 ng/mL (11~307 ng/mL)
Total bilirubin/direct bilirubin: 0.39/<0.1 mg/dL

Physical examination:
Pale face(+), Tachycardia (Heart rate: 110/min)

Diagnosis: Iron deficient anemia
Initial management: Collect stool sample and prescribe iron tablet 100 mg bid

7/4/2016

Stool examination showed a mild hemoccult-positive (1+).

Iron tablet 100 mg bid treatment

12/4/2016

13C urea breath test: positive
Upper GI endoscopy was arranged on 14/4/2016.

14/4/2016

Sudden onset of severe tenderness with involuntary guarding and rebounding pain involving the entire abdomen since early morning, 14/4/2016.
Interpretation of standing view and left lateral decubitus abdominal film detected free intraperitoneal air, and peritonitis through hollow organ perforation was confirmed.

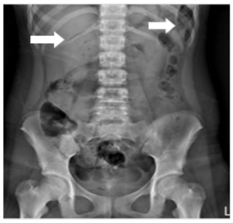

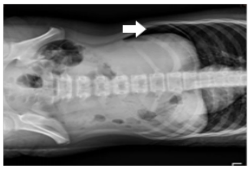

Emergent abdomen plain film examinations.
Surgical intervention was advised and in light of hemodynamic stability, a laparoscopic approach was performed. Laparoscopic findings:
1. A lot of dirty ascites and pus (left upper)
2. Debris and excised necrotic tissue (left lower)
3. Gastric ulcer perforation s/p repair (right)

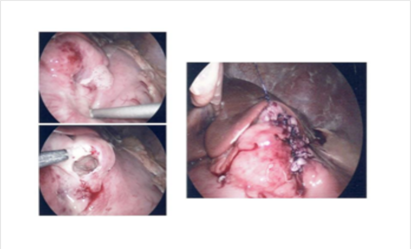

21/4/2016

Histology confirmed the diagnosis of extranodal marginal zone B-cell lymphoma of MALT type.

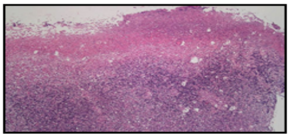

27/4/2016

Eradication of H.pylori was performed during 27/4/2016~11/5/2016

20/5/2016

Endoscopy was scheduled 4 weeks after operation and showed a deep and large ulcer over anterior wall of the body with convergence of thickened mucosal folds. Biopsy samples were again obtained and consistent with extranodal marginal zone lymphoma of MALT.

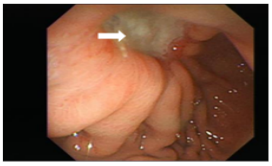

3/6/2016

Involved field radiation therapy was delivered to the stomach (30 Gy in 20 fractions given over 4 weeks).

25/8/2016

A follow-up endoscopy was performed at 4 months after operation, and showed a broad-based healed scar with rugae interruption (Figure 5b). The histological evaluation of biopsy specimen showed absent plasma cells and small lymphoid cells and complete histological remission was achieved at 2 months after radiotherapy.

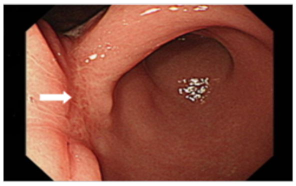

During a 1-year follow-up at our outpatient clinic, she has remained free of symptoms and without relapse.
